# Deep Learning with Automatic Data Augmentation for Segmenting Schisis Cavities in the Optical Coherence Tomography Images of X-Linked Juvenile Retinoschisis Patients

**DOI:** 10.3390/diagnostics13193035

**Published:** 2023-09-24

**Authors:** Xing Wei, Hui Li, Tian Zhu, Wuyi Li, Yamei Li, Ruifang Sui

**Affiliations:** Department of Ophthalmology, State Key Laboratory of Complex Severe and Rare Diseases, Peking Union Medical College Hospital, Chinese Academy of Medical Sciences, Peking Union Medical College, No. 1, Shuai Fu Yuan, Beijing 100730, China; weixingcocoa@163.com (X.W.); huihui2002_8@126.com (H.L.); zhutian1995@126.com (T.Z.); wuyili651@outlook.com (W.L.); liymdoct@163.com (Y.L.)

**Keywords:** optical coherence tomography, X-linked juvenile retinoschisis, image segmentation, deep learning, deep reinforcement learning

## Abstract

X-linked juvenile retinoschisis (XLRS) is an inherited disorder characterized by retinal schisis cavities, which can be observed in optical coherence tomography (OCT) images. Monitoring disease progression necessitates the accurate segmentation and quantification of these cavities; yet, current manual methods are time consuming and result in subjective interpretations, highlighting the need for automated and precise solutions. We employed five state-of-the-art deep learning models—U-Net, U-Net++, Attention U-Net, Residual U-Net, and TransUNet—for the task, leveraging a dataset of 1500 OCT images from 30 patients. To enhance the models’ performance, we utilized data augmentation strategies that were optimized via deep reinforcement learning. The deep learning models achieved a human-equivalent accuracy level in the segmentation of schisis cavities, with U-Net++ surpassing others by attaining an accuracy of 0.9927 and a Dice coefficient of 0.8568. By utilizing reinforcement-learning-based automatic data augmentation, deep learning segmentation models demonstrate a robust and precise method for the automated segmentation of schisis cavities in OCT images. These findings are a promising step toward enhancing clinical evaluation and treatment planning for XLRS.

## 1. Introduction

X-linked juvenile retinoschisis (XLRS; OMIM 312700) is an inherited disorder with an incidence rate ranging from 1:5000 to 1:25,000. It is regarded as one of the leading genetic causes of progressive retinal–vitreal degeneration in juveniles for males [1,2]. The characteristic features of the disease include varying degrees of central vision loss, radial streaks emanating from foveal schisis, splitting of the inner retinal layers in the peripheral retina, and a negative electroretinogram (ERG) prompted by a significant decrease in b-wave amplitude [1,3].

**Figure 1:**
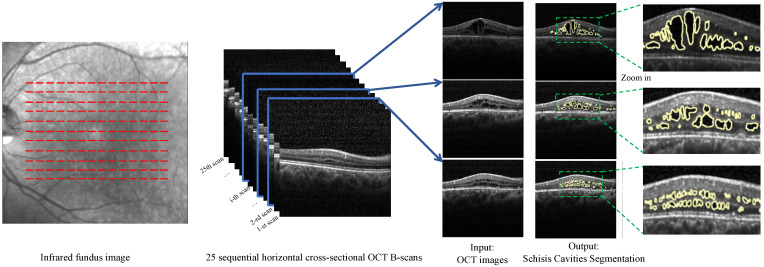
Demonstration of the diversity of schisis cavities in OCT images of XLRS patients. A patient’s fundus image is shown on the left side with red dashed lines indicating the locations of the 25 sequential horizontal cross-sectional OCT B-scans presented in the center panel. Three distinct B-scans (selected from the 25 scans) are displayed on the right to highlight the variance in the appearance of schisis cavities. Notably, despite being from the same patient, the schisis cavities exhibit significant heterogeneity due to variations in scan location. The corresponding expected schisis cavity segmentation and zoomed-in views of the cavities are also shown on the right side, with the targeted regions outlined by yellow lines.

The emergence of optic coherence tomography (OCT) has significantly transformed the diagnostic process for XLRS, with spectral domain OCT (SD-OCT) currently serving as the main diagnostic technique for this disorder. Notably, SD-OCT has proven vital for subsequent examinations and has settled the enduring controversy regarding the splitting of the specific retinal layer [4,5,6]. Indeed, SD-OCT plays a critical role in follow-up examinations and has resolved the long-standing debate about which retinal layer undergoes retinal splitting [7,8,9]. A characteristic feature of XLRS is the presence of schisis cavities within the retina, visible in OCT images [1,10,11,12,13] (Figure 1). These schisis cavities appear in various retinal layers and show considerable variability among different patients. As the disease progresses, the schisis cavities may change or potentially collapse, making the quantification of the schisis cavities area in OCT images a valuable metric for evaluating retinal structural changes and tracking disease progression [14,15]. This quantitative measure could further aid in assessing disease severity and inform the development of appropriate treatment strategies.

However, the manual segmentation of schisis cavities in OCT images is susceptible to deviations due to subjective interpretation, poor repeatability, and varied interobserver agreement [16,17]. Given the substantial volume of imaging data per patient, this method is also time consuming and unsuitable for clinical applications. Consequently, there is increasing interest in developing automated schisis cavity segmentation and measurement algorithms to enhance speed, reduce human effort, and improve accuracy. Several challenges impact the accuracy of these methods, including speckle noise due to the limited spatial bandwidth of the interference signals in OCT imaging, light absorption, and scattering in retinal tissue, these lead to intensity reduction in homogeneous areas with depth, and low contrast in certain OCT image regions caused by the optical shadowing of retinal blood vessels [16,18]. These challenges, compounded by motion artifacts and sub-optimal imaging conditions, necessitate a more efficient and precise approach.

Recent advancements in artificial intelligence (AI), particularly deep learning (DL), have brought promising developments due to the availability of expansive databases and powerful computing capabilities [19]. Ophthalmology, a field wherein diagnoses often rely on image analysis techniques, has emerged as a prominent area of AI research. DL has successfully segmented schisis cavities or fluids in OCT images for various common ocular diseases, such as age-related macular degeneration, retinal vein occlusion, diabetic macular edema, and ocular inflammation, with the popular frameworks convolutional neural network (CNN) and U-Net [16]. Alongside these advancements, several public datasets consisting of expert-annotated OCT images have been released, namely, the OPTIMA dataset [20], RETOUCH dataset [17], DME dataset [21], and UMN dataset [22].

Despite the availability of these resources, DL’s specific application to XLRS remains inadequately examined. A major hurdle is detecting the diverse schisis cavities characteristic of XLRS in OCT images (Figure 1), which is further complicated by a scarcity of relevant data. The OCT images vary greatly in appearance, contrast, and quality as they are acquired from different conditions and devices. Furthermore, the characteristics of schisis cavities vary significantly among patients, hampering the training and performance of DL models. To mitigate these challenges, the technique of data augmentation can be adopted to artificially expand the diversity and volume of the training dataset, thereby enhancing the performance of the segmentation model [23,24,25,26].

**Figure 2:**
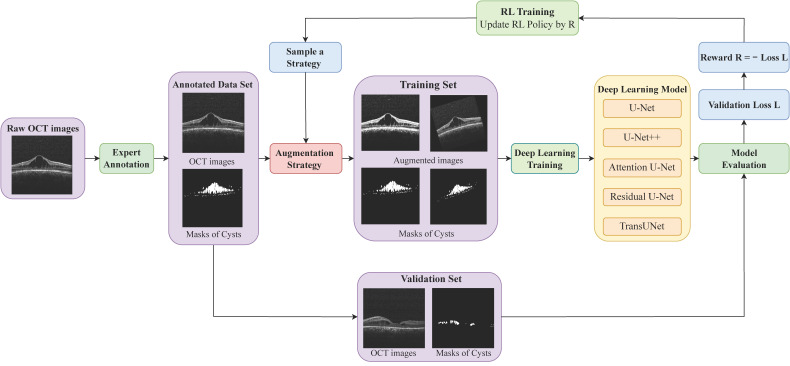
The pipeline of training workflow for automatic schisis cavity segmentation in OCT images. The raw OCT images are annotated by experts and processed via an RL-optimized augmentation strategy. The augmented dataset is utilized to train a DL model. Following this, the DL model’s loss on the validation set is computed and utilized as a reward signal to guide the subsequent updates to the RL’s policy.

In this study, we have designed a DL-based automatic schisis cavity segmentation pipeline in OCT images of XLRS patients, as shown in Figure 2. Notably, drawing inspiration from Cubuk et al.’s work [27] on image classification, we incorporated a reinforcement learning (RL)-based automatic data augmentation into our framework to boost the generalization and robustness of the DL segmentation model. Further, we trained five state-of-the-art DL models: U-Net [28], U-Net++ [29], Attention U-Net [30], Residual U-Net [31], and TransUNet [32]. We then conducted a quantitative comparison of their performance. Our findings demonstrate that U-Net++ exhibited superior performance in segmenting the schisis cavities in the OCT images for XLRS, as evidenced by its higher accuracy, Dice coefficient, precision, recall, specificity, and Jaccard index compared with the other models. Our automatic schisis cavity segmentation system achieved accuracy comparable to human-level evaluation. This strongly suggests the potential clinical applicability of our method in the domain of XLRS diagnosis and treatment.

## 2. Methods

### 2.1. Data Collection and Annotation

#### 2.1.1. Data Collection

This study involved patients diagnosed with XLRS from the Department of Ophthalmology at Peking Union Medical College Hospital (PUMCH) and Beijing Mei’ermu Hospital in Beijing, China. Prior to enrollment, informed consent was obtained from the patients or their legal guardians. The study was approved by the PUMCH Review Board (No. JS-2059) and adhered to the Ministry of Public Health of China’s Guidance on Sample Collection of Human Genetic Diseases and the Declaration of Helsinki.

We employed a dataset comprising 30 patients who were molecularly diagnosed with XLRS. OCT imaging was performed on both eyes of every patient. The imaging was executed using an SD-OCT device (Heidelberg Spectralis, Heidelberg Engineering, Heidelberg, Germany), capturing 25 horizontal cross-sectional B-scan images across a scanning area of 5.6 × 4.0 mm, with the fovea as the focal point. In total, the dataset included 1500 B-scan OCT images.

In the examined cohort, the onset age of the 30 patients was 14.76±15.75 years (IQR, 5.0–16.0; range, 1.0–58.0), and the visual acuity was 0.84±0.43 logMAR (IQR, 0.52–1.3; range, 0.10–2.70). The schisis cavities were observed in the ganglion cell layer, inner plexiform layer, inner nuclear layer, outer plexiform layer, and outer nuclear layer in the OCT images, with respective prevalence rates of 10.9%, 1.6%, 100%, 21.9%, and 31.2%.

#### 2.1.2. Manual Annotation

The labeling of schisis cavities in OCT images was manually carried out using the ZEISS APEER platform (www.apeer.com). Two ophthalmologists with over five years of experience conducted these annotations between January and March 2023.

To minimize inter-reader variability, we established an annotation protocol prior to the process. This protocol is based on the clinical characteristics of XLRS patients in OCT and also references the following works [20,33]. The protocol considers the following: cyst regions should be marked only when the reader is confident they genuinely represent cysts. These regions typically possess visible boundaries, making them distinctly distinguishable from non-cyst areas. In terms of their shape, while cysts predominantly display a circular or oval form, their proximity to other cysts can occasionally lead to areas with varied shapes. Furthermore, cysts are generally present in consecutive B-scans, so it is imperative for readers to verify their existence in both following and preceding scans. Clinically speaking, cysts are most commonly observed in the inner nuclear layer, outer plexiform layer, and outer nuclear layer, prompting readers to particularly focus on these regions during the annotation process.

Each ophthalmologist annotated half of the dataset. After their individual annotations, they reviewed each other’s results. In cases of discrepancies, they discussed and adjusted the annotations based on mutual agreement. The platform APEER supports revision post-annotation. Consequently, we have a single, consolidated version of annotations for each image. The manual annotation of schisis cavities in OCT images provided the foundational basis for the subsequent deep learning training.

### 2.2. Deep Learning Model for Schisis Cavity Segmentation in OCT

#### 2.2.1. Neural Network Architecture

**Figure 3:**
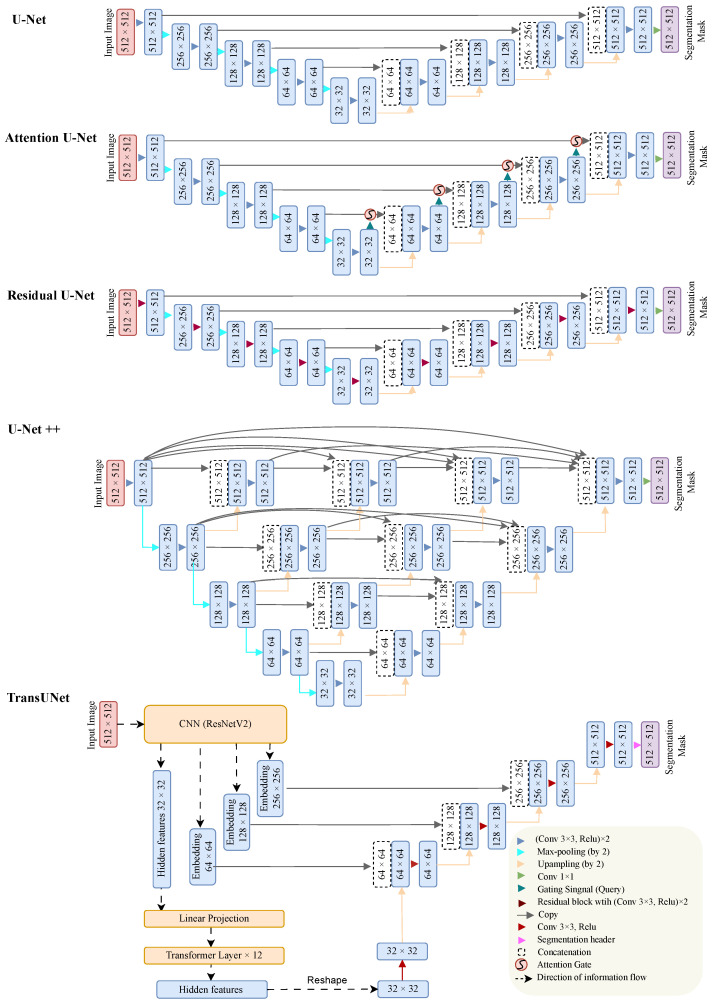
The neural network architectures of U-Net, Attention U-Net, Residual U-Net, U-Net++, and TransUNet. The numbers within the blue squares represent the data dimensions (W×H). For more detailed information about the components illustrated in the figure, such as the attention gate, please refer to the original papers [28,29,30,31,32].

In our endeavor to segment the schisis cavities, we leveraged five advanced deep learning models: U-Net, U-Net++, Attention U-Net, Residual U-Net, and TransUNet. These model architectures are visually illustrated in Figure 3. U-Net, a specific variety of CNN, is recognized for its distinctive U-shaped architecture. This structure comprises a contracting path (or encoder) for capturing context and a corresponding expanding path (or decoder) for precise localization. Attention U-Net, a refinement of the traditional U-Net, introduces an attention gate (AG) into the model’s skip connections. The AG allows the model to concentrate on certain specific regions of the input image which are particularly pertinent to the task in question. Residual U-Net is designed with the objective of enhancing the flow of gradients during the model training process by incorporating a residual module with a shortcut bypassing two convolutional layers. U-Net++, another variant of the original U-Net, promotes feature aggregation across different semantic scales by re-engineering the simple skip connections of U-Net into nested and dense connections. TransUNet stands out by fusing the vision transformer (ViT) [34] into the U-Net architecture. It employs a CNN to generate a feature map for the input. The model’s prominent advantage is its capacity to encapsulate global contextual information within images, a feature that could be significantly advantageous for complex segmentation tasks.

#### 2.2.2. Deep Neural Network Training

The pipeline for this research is outlined as follows and is further visualized in Figure 2. We adopted a *k*-fold cross-validation strategy (where k=5) to partition the annotated dataset. For each split, the data are divided into a training portion (comprising 4 folds, 80% of the entire dataset) and a test set (1 fold or 20%, the entire dataset). Then, we directly randomly split 87.5% of the training portion (70% of the entire dataset) as the training set of our deep learning model. The residual 12.5% of the training portion (which is 10% of the entire dataset) is marked as validation data that is specifically utilized to refine the augmentation strategy (elaborated on in the subsequent section). Importantly, the processes of folding and splitting were implemented at the patient level, thus ensuring that B-scans from identical OCT volumes were not included in both the training and testing subsets simultaneously.

Each OCT image utilized in this study was transformed into a 1-channel grayscale image with dimensions of 1×512×512 pixels. The schisis cavity mask was represented using one-hot encoding, with dimensions of 2×512×512. A data augmentation process was implemented on the training set, following the strategy derived from RL, the details of which will be elaborated on in Section 2.3. The augmented training set was subsequently input into the DL model.

Our research incorporates a hybrid loss function during the training process. This function amalgamates two conventional loss functions that are frequently applied in medical image segmentation: cross-entropy (CE) and the Dice–Sorensen coefficient (DSC) loss functions. The combined loss function utilized can be expressed mathematically as

(1) $$L_{used}=\frac{1}{2}L_{CE}+\frac{1}{2}L_{Dice}.$$

The CE is a variant of the Kullback–Leibler (KL) divergence that measures the divergence between two probability distributions. In the context of segmentation tasks, the CE can be described as follows:

$$\begin{array}{c} L_{CE}=-\frac{1}{N}\sum_{i=1}^{C}\sum_{j=1}^{N}p_{j}^{i}\log q_{j}^{i}. \end{array}$$

Here, $p_{j}^{i}$ symbolizes the binary indicator of the class label *i* for voxel *j* from the ground truth, while $q_{j}^{i}$ corresponds to the estimated segmentation probability.

The DSC is commonly employed to determine the similarity between two data samples. The DSC can be expressed as follows:

$$\begin{array}{c} L_{Dice}\left(y,\hat{y}\right)=1-\frac{2y\hat{y}+\alpha }{y+\hat{y}+\alpha }. \end{array}$$

In this equation, *y* and $\hat{y}$ signify the actual and predicted values generated by the model, respectively. The variable α maintains the function’s definition in edge cases, such as when $y=\hat{y}=0$.

### 2.3. Data Augmentation

#### 2.3.1. Problem Formulation

Data augmentation serves as a strategy to artificially augment the volume and diversity of the training dataset, thereby significantly enhancing the performance and generalization of the segmentation model. The auto-augmentation strategy search aims to discover an optimal augmentation strategy, consisting of multiple image transformation operations.

Given a loss function L and a training set $D^{train}$, a general machine learning algorithm $A:D^{train}\to M$ strives to derive a model *M* with parameter ω from the model space M such that

(2) $$A\left(D^{train}\right)=\underset{M_{\omega }\in M}{\mathrm{argmin}}L\left(M_{\omega },D^{train}\right).$$

Despite this, the trained model $M_{\omega }$ may encounter issues with generalization errors. To address this, the aim of the augmentation strategy search is to identify an optimal augmentation strategy F∈F that results in a model $M_{\omega^{*}}$ where the generalization error on the validation set is minimized.

(3) $$\begin{array}{cc} F^{*} & =\underset{F\in F}{\mathrm{argmin}}L\left(A\left(F\left(D^{\mathrm{train}}\right)\right),D^{\mathrm{valid}}\right) \end{array}$$

(4) $$\begin{array}{cc} \; & =\underset{F\in F}{\mathrm{argmin}}L\left(\underset{M_{\omega }\in M}{\mathrm{argmin}}L\left(M_{\omega },F\left(D^{\mathrm{train}}\right)\right),D^{\mathrm{valid}}\right), \end{array}$$

where $D^{\mathrm{train}}⋂D^{\mathrm{valid}}=\emptyset$. This optimization procedure aims to find the optimal *F* that delivers the best performance for the validation data, thereby improving the model’s generalization capabilities. This situation represents a bilevel optimization problem [35]. At the outer level, a search is conducted for an augmentation strategy *F*, which facilitates the attainment of the best-performing model $M_{\omega^{*}}$.

#### 2.3.2. Search Space

We explicitly define the augmentation strategy *F* and its corresponding search space F. The input domain of images is denoted as X. An image transformation operation is represented as o∈O:X→X, functioning within the domain X. Each operation *o* is associated with a magnitude μ and is implemented with a probability ϕ, represented as o(x;μ,ϕ). The image transformations o(x;μ,ϕ) utilized in this study were sourced from the Python Imaging Library (PIL). Our search included operations such as ShearX(Y), TranslateX(Y), Rotate, Color, Posterize, Solarize, Contrast, Sharpness, Brightness, AutoContrast, Equalize, Invert, Horizontal Flip, and Vertical Flip. The mask image should be transformed in accordance with the transformation strategy that was applied to its corresponding original image. The details of the operations and their magnitude are listed in Table 1.

The augmentation strategy is defined as $F=\{{\tilde{f}}_{1},{\tilde{f}}_{2},\cdots ,{\tilde{f}}_{5}\}$, encompassing five sub-strategies. A sub-strategy, denoted $\tilde{f_{i}}$, incorporates two successive image transformation operations, expressed as

$$\tilde{f_{i}}=o_{1}\left(x;\mu_{1},\phi_{1}\right)\circ o_{2}\left(x;\mu_{2},\phi_{2}\right).$$

**Figure 4:**
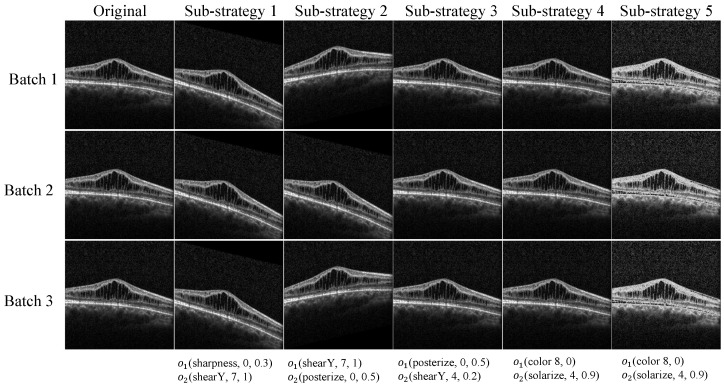
Illustration of results using a sampled augmentation strategy. The image depicts the outcome of our image augmentation strategy applied to an original training image. The employed strategy encompasses five sub-policies. Within each mini-batch, we randomly choose one sub-policy and apply it to the image for the neural network training. Each sub-policy consists of two operations, each associated with two numerical values: the magnitude of the operation and the probability of its application. Thus, an operation may not always be applied within a given mini-batch due to the probability attached to its execution.

Each image in the dataset is sequentially processed via this augmentation strategy, where each sub-strategy *i* is applied with a specific magnitude $\mu_{i}$ and a corresponding probability $\phi_{i}$. An example of a strategy with five sub-strategies within our search space is illustrated in Figure 4. The search space encompasses 16 operations, each possessing a predefined range of magnitudes. To maintain stability during the search process, we have confined the magnitude μ of each operation within a particular range. Given that the majority of operation magnitudes are non-differentiable, we have partitioned the range into ten equally distributed values. Similarly, the probability of executing an operation is discretized into eleven equally distributed values. Consequently, identifying each sub-policy equates to a search problem within a space containing (${16\times 10\times 11)}^{2}$ potential possibilities. For operations that do not necessitate magnitude differentiation, such as AutoContrast, the sampled magnitude parameters are disregarded. Our goal is to simultaneously discover five distinct sub-policies to augment diversity, effectively enlarging the search space to approximately (${16\times 10\times 11)}^{10}$ possibilities, which surpasses $10^{32}$.

#### 2.3.3. Optimizing the Augmentation Strategy with Reinforcement Learning

We utilized RL to solve the optimal strategy search problem outlined in Equation (4). The RL agent’s action corresponds to the augmentation strategy *F* applied to the DL model. The state at a given time *t* is represented as the history of actions, denoted as a list of actions $a_{(t-1):1}$. We formulate the agent’s policy $\pi_{\theta }$ as an augmentation strategy controller, which is modeled as a recurrent neural network (RNN) to encapsulate all historical data effectively. The reward function of the RL is defined as the negative evaluation loss of the DL model $M_{\omega }$ on the validation set, as depicted in our work’s pipeline (Figure 2), and represented as

(5) $$R=-L(M_{\omega },D^{valid}).$$

The objective of the RL agent is to identify an optimal policy $\pi_{\theta^{*}}$ that maximizes the expected reward, denoted by J(θ), over a specified time horizon T. This goal is aligned with the outer level optimization problem delineated in Equation (4), minimizing the validation loss of the DL model.

(6) $$J\left(\theta \right)=E_{\pi_{\theta }\left(a_{1:T}\right)}\left[R\right].$$

Then, the proximal policy optimization (PPO) algorithm [36] is employed to iteratively fine-tune the policy parameters, θ. This algorithm facilitates the RL policy’s adjustment process, ultimately aiding in identifying the optimal augmentation policy for the DL model.

(7) $$\nabla_{\theta }J\left(\theta \right)=\nabla_{\theta }\log\pi_{\theta }\left(a_{t}|a_{(t-1):1}\right)R.$$

The comprehensive training protocol, which includes the DL model and the RL agent, is elaborated in Algorithm 1. This algorithm is designed to identify the optimal DL model and the best RL policy. The algorithm’s overarching structure consists of an outer loop for RL training and two nested loops for DL training. In each iteration from 1 to T in the RL training phase the algorithm samples an augmentation strategy *F* derived from the RL policy $\pi_{\theta }$. Subsequently, in the DL training phase, the algorithm undertakes multiple epochs within the nested loops. For every batch in each epoch, a sub-strategy *f* is uniformly selected from *F*, and then mini-batch data $D_{i}^{train}$ are applied to the sampled augmentation sub-strategy *f*. The augmented minibatch data $D_{i}^{aug}$ are used to update the DL model parameters ω. Once the DL training phase is complete, the algorithm evaluates the loss of the DL model on the validation dataset. This loss value is subsequently used to define the reward for the RL agent. Finally, the RL policy parameters θ are updated by leveraging the calculated reward *R* and the policy gradient. This entire process is iteratively performed until a defined termination condition is met. Once the search procedure is concluded, we consolidate the sub-strategies from the top five strategies into one encompassing strategy, which consists of 25 sub-strategies in total. This combined strategy is then leveraged to train the final DL models.
**Algorithm 1** Automated augmentation via DRL for DL segmentation models**Initialization:** Segmentation DL model $M_{\omega }$, RL augmentation policy controller $\pi_{\theta }$ **Input:** Training dataset $D_{train}$, Validation dataset $D_{valid}$ **Output:** $\omega^{*}$, $\theta^{*}$  1: **for** 1≤t≤T **do**  2:        Sample an augmentation strategy from RL policy $\pi_{\theta }$, $F∼\pi_{\theta }$  3:        **for** 1≤e≤E **do**  4:            **for** 1≤i≤B **do**  5:               Choose a sub-strategy *f* uniformly from *F*, f∼F  6:               Augment the mini-batch data by *f*, $D_{i}^{aug}=f(D_{i}^{train}$)  7:               Update ω by ${\mathrm{argmin}}_{M_{\omega }\in M}L\left(M_{\omega },D_{i}^{aug}\right)$  8:            **end for**  9:        **end for** 10:       Evaluate the loss of the DL model on the validation set, $Loss=L(M_{\omega },D^{valid})$ 11:       Define the reward for RL agent, $R_{t}=-Loss$ 12:       Update the policy parameters θ, by reward *R* and the gradient $\nabla_{\theta }J\left(\theta \right)$ 13: **end for**

The policy controller RNN is implemented as a single-layer long short-term memory (LSTM) model, comprising 100 hidden units per layer. The LSTM’s prediction layers consist of two fully connected layers, which offer softmax predictions for each operation, necessitating an operation type, magnitude, and probability. In total, the LSTM requires 30 predictions to configure five sub-strategies. Each of these sub-strategies is composed of two operations, where every individual operation demands the specification of an operation type, a corresponding magnitude, and an associated probability.

### 2.4. Evaluation Metrics

The performance of the segmentation algorithms is evaluated based on six different metrics, including accuracy, precision, recall, Dice coefficient, specificity, and the Jaccard index.

**Precision/recall:** Precision and recall are computed either for each individual class or collectively and are defined as follows:

(8) $$\begin{array}{c} \mathrm{Precision}=\frac{\mathrm{TP}}{\mathrm{TP}+\mathrm{FP}},\;\mathrm{Recall}=\frac{\mathrm{TP}}{\mathrm{TP}+\mathrm{FN}}, \end{array}$$

where TP represents true positive, FP denotes false positive, and FN signifies false negative. In the realm of segmentation, recall is alternatively referred to as sensitivity, and calculates the fraction of correctly labeled foreground pixels while ignoring background pixels.

**Accuracy:** Often known as class average accuracy, this metric quantifies the proportion of correctly classified pixels relative to the total number of pixels in each class. Its formulation is as follows:

$$\begin{array}{c} Acc=\frac{1}{k}\sum^{k}j=1\frac{p_{jj}}{g_{j}}, \end{array}$$

where $p_{jj}$ represents the number of correctly classified pixels for class *j* and $g_{j}$ denotes the total number of pixels in the ground truth for class *j*. However, the frequent class imbalance observed in medical datasets may adversely influence the performance.

**Jaccardindex:** Also known as the intersection over union (IoU), this measure calculates the degree of overlap between the predicted segmentation mask and the ground truth. Its mathematical representation is as follows:

$$\begin{array}{c} J\left(A,B\right)=\frac{\left|A\cap B\right|}{A\cup B}=\frac{\mathrm{TP}}{\mathrm{TP}+\mathrm{FP}+\mathrm{FN}}. \end{array}$$

In this equation, *A* and *B* symbolize the ground truth and the predicted segmentation, respectively.

**Dicecoefficient:** This metric, frequently used in medical image analysis, computes the ratio of twice the area of overlap between the ground truth (*G*) and the predicted (*P*) maps to the sum of pixels in both areas. It is defined as:

$$\begin{array}{c} Dice=\frac{2|G\cap P|}{\left|G|+|P\right|}. \end{array}$$

In the case of binary segmentation maps, the Dice score prioritizes the accuracy of the foreground pixels while penalizing incorrect predictions.

### 2.5. Implementation Details

Several batch sizes for the DL model were tested, specifically 16, 8, 4, and 2, with the most effective results discussed herein. The number of training epochs *E* for the DL was set to 200. Models such as U-Net, U-Net++, Attention U-Net, and Residual U-Net utilized the Adam optimizer with a learning rate of 1 × $10^{-4}$. We also employed an adaptive learning rate strategy, ReduceLROnPlateau, with a hyperparameter factor set at 0.5 and a patience level of 10. For TransUNet, the stochastic gradient descent (SGD) optimizer was used with a learning rate of 0.01, momentum of 0.9, and weight decay of 1 × $10^{-4}$.

For the RL agent, the policy gradient algorithm was employed with a learning rate of 4 × $10^{-4}$ and an entropy penalty of weight 1 × $10^{-5}$. The controller weights were uniformly initialized between −0.1 and 0.1, with the time horizon *T* set to 1000.

The computational experiments were executed using Pytorch 2.0.1 with CUDA 11.8, Python 3.9.13, and hardware comprising eight NVIDIA RTX A5000 GPUs (Nvidia Corporation, Santa Clara, CA, USA), dual AMD EPYC 7662 CPUs (Advanced Micro Devices, Inc., Santa Clara, CA, USA), and 256 GB of RAM.

## 3. Results

We optimized the augmentation strategy using RL separately for U-Net, U-Net++, Attention U-Net, Residual U-Net, and TransUNet. Table 2 presents the results of the automated augmentation strategies optimized using RL for U-Net++. This includes a breakdown of 25 sub-strategies. Notably, the most frequently occurring transformations are Contrast/AutoContrast, Equalize, Sharpness, Brightness, and Solarize. While other models also follow similar policies, the parameters vary. Interestingly, geometric transformations such as ShearX, ShearY, and Rotation are seldom found in the final strategy automatically determined using RL.

**Figure 5:**
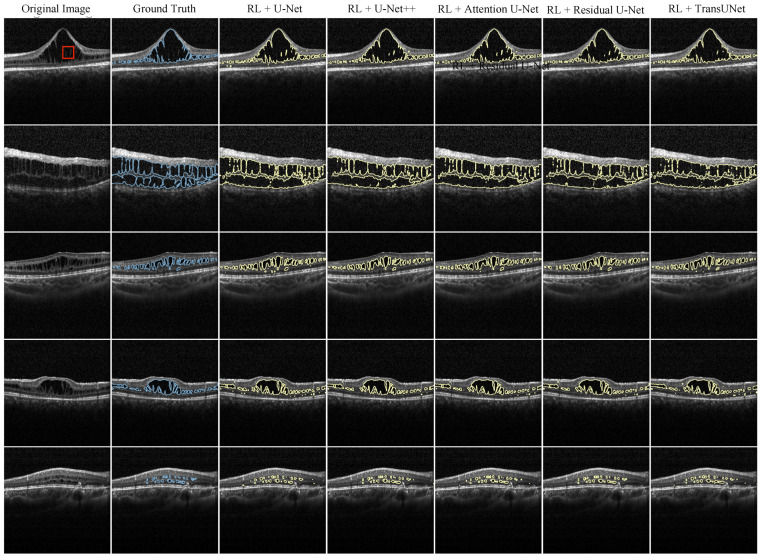
Segmentation results from U-Net, U-Net++, Attention U-Net, Residual U-Net, and TransUNet with RL data augmentation on the test set are presented. The first column showcases the original images, while the second column displays the ground truth, annotated by an expert. The subsequent columns represent the segmentation results obtained from the respective DL models. Expert annotations are highlighted in blue within the second column, and the segmentation results from the other models are outlined with a yellow line. The red square in the top left corner of the subfigure highlights a specific tissue that was not marked for exclusion by the experts but correctly identified by DL models.

The segmentation results in the test set are shown in Figure 5. It was observed that with the help of an RL-optimized augmentation strategy, all DL models delivered admirable results. Notably, in the first row, the boundary of schisis cavities identified by the DL models occasionally exceeded the expert-provided annotations. Within the largest schisis cavities in the first row’s original image, there exist regions that should be excluded due to the presence of certain tissue (indicated by red squares in the original image). These areas were not marked as exclusions by the experts but were correctly identified by all DL models. This highlights the considerable potential of our methodology for clinical applications, suggesting an improved level of accuracy in the measurement of schisis cavities.

**Figure 6:**
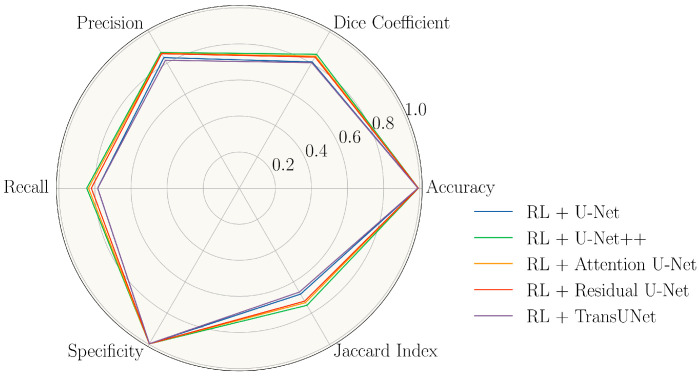
Performance comparison of five deep learning models with RL data augmentation: U-Net, U-Net++, Attention U-Net, Residual U-Net, and TransUNet, across six evaluation metrics on the test dataset. The metrics (plotted radially) include accuracy, Dice coefficient, precision, recall, specificity, and Jaccard index, which provide a comprehensive performance view. Each model is represented by a unique polygon in the plot that extends from the center to the measured value for each metric. A higher value signifies better performance for the respective metric.

To further quantitatively assess and compare the performance of the deep-learning-based segmentation approach across five distinct models, we leveraged six different metrics, including accuracy, precision, recall, Dice coefficient, specificity, and the Jaccard index. As highlighted in the Introduction, there is a notable absence of models specifically tailored for XLRS in the literature due to the variability observed across different patients and the lack of sufficient data. So, we used RetiFluidNet [37] as a baseline for comparison, which was originally developed for fluid segmentation in OCT targeting age-related macular degeneration. In their original work, the augmentation strategy of RetiFluidNet comprised random selections from translations, rotations, contrast adjustments, and mirroring. We maintain settings consistent with this in our experiment. An ablation study was also conducted by removing the RL component to illustrate the advantages of using RL for data augmentation. Table 3 presents the mean and standard deviation of the six metrics of the five models with and without RL data augmentation across the test sets. In addition to the tabular representation, we also depicted the performance of the five models with RL augmentation using a radar plot (Figure 6).

Our results show that U-Net++ outperforms the other models across most evaluation metrics when RL augmentation is applied, achieving an accuracy of 99.27%, Dice coefficient of 85.68%, precision of 86.91%, recall of 84.52%, specificity of 99.66%, and a Jaccard index of 75.00%. The baseline RetiFluidNet with random augmentation performs slightly better than other U-Net variants. However, it still lags behind the U-Net++ integrated with RL augmentation. This indicates that U-Net++ demonstrates superior performance in the task of schisis cavity segmentation, particularly when supplemented with RL data augmentation.

**Figure 7:**
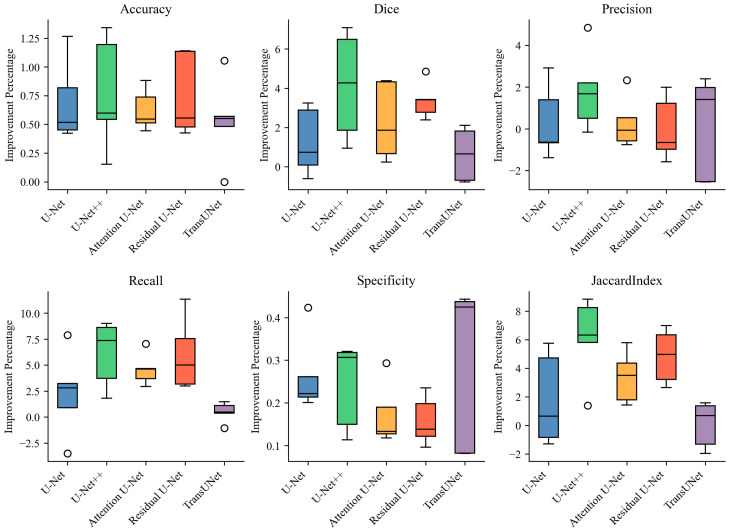
Performance enhancement in five deep learning models using RL augmentation on test data: U-Net, U-Net++, Attention U-Net, Residual U-Net, and TransUNet. Six evaluation metrics obtained from cross-validation are considered.

We further compare the average improvements achieved by the models with RL data augmentation in Figure 7. It is apparent that utilizing the RL augmentation approach leads to a notable increase across all six evaluation metrics, particularly in the Dice coefficient, recall, and Jaccard index. More specifically, significant performance enhancements are seen in U-Net++, Attention U-Net, and Residual U-Net when applying RL data augmentation, with improvements ranging from 2% to 6% in the Dice score, recall, and Jaccard index.

A statistical analysis using the *t*-test was also conducted. Table 4 presents the *p*-values for the six metrics across five DL models on the test set for the cases with and without RL augmentation. With data augmentation through RL, all five models exhibited significant enhancements in accuracy. Both UNet++ and Residual U-Net demonstrated notable improvement in the Dice score. Meanwhile, Residual U-Net and TransUnest significantly improved in terms of recall. As for specificity, UNet, UNet++, Attention U-Net, and TransUNet all showed marked advancements. Furthermore, both UNet++ and Residual U-Net documented significant improvements in the Jaccard index. These results collectively highlight the utility and efficacy of RL-based data augmentation for boosting the segmentation performance of deep learning models.

## 4. Discussion

This study delved into the effectiveness of DL models, including U-Net, U-Net++, Attention U-Net, Residual U-Net, and TransUNet, in the segmentation of schisis cavities in the OCT scans of patients with XLRS, as depicted in Figure 2. Segmenting schisis cavities in OCT images presents a challenge that can be addressed by employing data augmentation to expand the diversity of the training data. This approach enhances the model’s robustness, reduces overfitting, and elevates accuracy. We defined a search space for a given dataset and DL model, and employed deep RL to determine the optimal policy. In particular, the PPO algorithm was used to achieve the highest validation dataset accuracy. This optimal strategy can be implemented in the training set to bolster the performance of the DL model in the segmentation task. The RL-based data augmentation technique considerably improved the robustness and generalizability of the DL model used for schisis cavity segmentation. The RL methodology generalizes and applies the findings of Cubuk et al.’s study [27] on image segmentation tasks.

Historically, numerous studies have delved into data augmentation for medical image segmentation [23,24,25,26]. These research efforts underscore the importance of maintaining anatomical and structural integrity in medical images through transformation-based image augmentation, especially for medical image segmentation tasks. Notable advances have been made in the automation of determining the optimal data augmentation approach, evidenced by its successful application in classification tasks on ImageNet [27,38] and the Medical Segmentation Decathlon challenge [39,40]. The augmentation strategy adopted in our work aligns with these findings. Another avenue of research suggests that even if certain transformations might distort anatomical information in an image, they can still bolster the model’s generalizability. This is evident with methods like elastic deformation, which have found application in many medical image segmentation tasks. We aim to delve into these strategies in our subsequent studies [41,42,43].

Image segmentation and image classification are profoundly interconnected disciplines within the broader field of computer vision. Fundamentally, image segmentation can be conceptualized as the fine-grained classification of individual pixels into designated categories within a given image. This association leads to an academically compelling inquiry: Can the efficacy of a data augmentation strategy, when proven in image classification paradigms, be seamlessly extrapolated to image segmentation endeavors? Our preliminary tests, wherein we directly applied the augmentation policy from [27] to our segmentation problem, provided an unexpected answer. We found instances where this augmentation negatively impacted our model’s performance. Consequently, we developed our own augmentation policy, specifically tailored to meet our project’s demands. For the OCT segmentation task, as exemplified by U-Net++ in Table 2, we noticed that the prevalent operations are similar to those in [27] but with varied parameters. This suggests that while operation parameters are crucial, certain similarities persist across different tasks. Another notable point raised in [27] is the concept of augmentation strategy transferability across datasets and models. This idea resonates with us, and we are keen to explore its application in the realm of image segmentation in our future studies.

For the OCT schisis cavities segmentation task, our RL policy identified that the most frequently occurring transformations were Contrast, Equalize, Sharpness, and Brightness. In contrast, geometric transformations like ShearX and ShearY were rarely observed in our final strategy. The primary goal of data augmentation is to enhance the model’s generalizability on the test set. Intuitively, after augmentation, if the training data distribution closely resembles the test data distribution, the performance will be improved more. In fact, due to varying protocols and operator differences in OCT scans, images frequently show changes in contrast and brightness instead of geometric alterations like shear. Hence, the identified augmentation strategies are more likely to produce images that, while distinct from the original, align more closely with real-world scan scenarios, i.e., the test data. This alignment can explain the observed improvement in performance and the efficacy of our chosen strategy. To delve deeper into the influence of each transformation, future endeavors could include conducting an ablation study to isolate the impact of each transformation to discern the most effective one. Additionally, leveraging algorithms from causality in interpretable RL could provide insights into the policy and answer why specific augmentation strategies are selected [44,45].

Regarding neural network architectures, the U-Net model has made considerable strides over predecessors like CNN, especially in image segmentation tasks. U-Net is useful in various medical applications, such as CT scans [46,47,48], MRIs [49,50], X-rays [51,52], OCT [53,54,55], and PET [56,57]. The U-Net architecture has proven instrumental in the field of medical image segmentation, spawning numerous model variants that advance the current state of the art. When comparing the models used in our study, U-Net++ outperformed the others, with superior accuracy, Dice coefficient, precision, specificity, and Jaccard index. With the RL data augmentation, U-Net-related architectures achieved Dice coefficients ranging from 0.80 to 0.84 and showed excellent performance in evaluation metrics, highlighting the benefits of the U-Net structure.

The baseline model RetiFluidNet is designed to assess retinal fluid in OCT caused by age-related macular degeneration. This condition shares similar characteristics with retinal schisis in XLRS. The model optimizes hierarchical representation learning of textural, contextual, and edge features by leveraging a new self-adaptive dual-attention module. Additionally, it incorporates multiple self-adaptive attention-based skip connections and a novel multi-scale deep self-supervision learning scheme. In simulations, RetiFluidNet outperforms the traditional U-Net, benefiting from its design. However, given that the RetiFluidNet’s settings rely on random augmentation strategies, its performance still falls short compared to our enhanced U-Net++ with RL automatic augmentation. Considering the computational costs, our RL augmentation policy training indeed requires more computational resources to pinpoint the optimal augmentation strategy when compared to the baseline. Still, the results showcase superior performance. There is an inherent trade-off between training time and performance. As previously mentioned, the transferability of the augmentation strategy could be beneficial to speed up the training. We aim to delve deeper into this in our future work.

Manual human segmentation of the schisis cavities in OCT images is unfeasible. Therefore, DL presents a consistent and scalable solution that could potentially facilitate innovative approaches to disease management, clinical trial evaluation, and scientific exploration. The flexibility of our DL-assisted segmentation is particularly significant in the context of XLRS OCT imaging. In the pursuit of understanding the disease’s natural progression and investigating the potential of gene therapy, the current research critically requires robust clinical markers to accurately measure disease progression and predict outcomes. Traditional OCT studies for XLRS have primarily focused on total retinal thickness, influenced by both thinning due to outer retinal atrophy and thickening from schisis cavities. It becomes challenging to distinguish whether a decrease in total retinal thickness is due to a reduction in schisis cavities or due to the loss of photoreceptors [14,15]. With the assistance of our segmentation results, the area of the schisis cavities can be accurately evaluated, providing a more precise metric for tracking structural changes throughout the disease’s progression. The automated and swift computation capabilities of the DL approach promote routine, efficient assessments of schisis cavity changes during disease progression.

Despite our promising results, there are several limitations to this study. Firstly, our sample size of 30 patients, although representative for a rare disease, might not be enough to generalize our findings to the entire XLRS patient population. Secondly, while we used data from one OCT device, incorporating additional devices could further validate our findings and enhance the generalizability of our models. Thirdly, while our current DL models and other schisis cavity segmentation studies do not leverage the correlation between scans, investigating novel network structures to enhance segmentation precision represents a promising future research direction.

## 5. Conclusions

In this study, we present an automated pipeline for segmenting schisis cavities in the OCT images of XLRS patients, utilizing DL models. These models are enhanced with data augmentation strategies automatically driven by RL. The segmentation results produced by our model exhibited a degree of accuracy comparable to that of human experts. Among the examined models, U-Net++ showed superior performance over other U-Net-related network architectures, underscoring its capability to effectively identify schisis cavities in OCT images. The RL-guided automatic data augmentation boosted the versatility and robustness of our DL model designed for schisis cavity segmentation. These findings suggest that U-Net++ could be particularly well suited for clinical situations that necessitate the accurate segmentation of schisis cavities in OCT images of XLRS patients. Additionally, RL-enhanced automatic data augmentation can elevate the performance of DL models in segmentation tasks. Moreover, the DL-based segmentation methods significantly improve clinical assessment and the formulation of treatment plans for XLRS patients. Future research endeavors will explore the transferability between different policies and models in the OCT segmentation domain, and the influence of annotation variability on segmentation outcomes.

## Figures and Tables

**Table 1:** Image transformations and magnitude ranges.

Operation	Magnitude	Operation	Magnitude
ShearX(Y)	[−0.3, 0.3]	Sharpness	[0, 0.9]
TranslateX(Y)	[−150, 150]	Brightness	[0, 0.9]
Rotate	[−30, 30]	AutoContrast	-
Color	[0.1, 0.9]	Equalize	-
Posterize	[4, 8]	Invert	-
Solarize	[0, 256]	Horizontal Flip	-
Contrast	[0, 0.9]	Vertical Flip	-

**Table 2:** The data augmentation strategy optimized by RL for UNet++. The first parameter is the level in the parameter range, and the second one is the probability.

Sub-Strategy Index	Operation 1	Operation 1 Parameter	Operation 2	Operation 2 Parameter
1	Sharpness	2, 0.5	AutoContrast	-, 0.4
2	Equalize	-, 0.9	TranslateY	4, 0.7
3	Rotate	3, 0.5	Posterize	9, 0.4
4	Sharpness	8, 0.8	Invert	-, 0.3
5	Contrast	8, 0.4	TranslateY	3, 0.8
6	Brightness	4, 0.7	Equalize	-, 0.2
7	ShearX	1, 0.2	Posterize	3, 0.9
8	Solarize	2, 0.7	Sharpness	7, 0.2
9	AutoContrast	-, 0.4	Horizontal Flip	-, 0.4
10	TranslateY	4, 0.7	Sharpness	2, 0.8
11	Solarize	7, 0.2	Brightness	2, 0.6
12	Invert	-, 0.8	Solarize	7, 0.2
13	Sharpness	3, 0.6	Rotate	8, 0.3
14	Equalize	-, 0.4	Contrast	8, 0.4
15	Solarize	7, 0.2	Brightness	5, 0.2
16	Equalize	-, 0.9	Contrast	3, 0.6
17	Sharpness	2, 0.2	Invert	-, 0.7
18	Contrast	3, 0.3	Invert	-, 0.3
19	Sharpness	2, 0.9	TranslateY	7, 0.7
20	AutoContrast	-, 0.8	Posterize	5, 0.2
21	ShearX	1, 1	Rotate	7, 0.7
22	Contrast	-, 0.3	Equalize	-, 0.2
23	Equalize	-, 0.3	Sharpness	8, 0.8
24	TranslateY	3, 0.1	Equalize	-, 0.9
25	Solarize	4, 0.1	Contrast	2, 0.5

**Table 3:** The evaluation of DL models for schisis cavity segmentation in the test set. The values presented in the table represent the mean and standard deviations of each metric derived from a five-fold cross-validation. The top-performing results are highlighted in bold.

Model	Accuracy	Dice	Precision	Recall	Specificity	Jaccard Index
U-Net	98.34 ± 0.60	79.50 ± 2.50	83.26 ± 2.83	76.21 ± 4.26	99.34 ± 0.19	66.04 ± 3.48
RL + U-Net	99.03 ± 0.20	80.77 ± 3.12	83.59 ± 5.10	78.48 ± 5.36	99.60 ± 0.08	67.84 ± 4.47
U-Net++	98.50 ± 0.52	81.55 ± 1.84	85.09 ± 3.75	78.41 ± 2.55	99.42 ± 0.15	68.88 ± 2.62
RL + U-Net++	**99.27** ± 0.14	**85.68** ± 2.13	**86.91** ± 1.80	**84.52** ± 3.02	**99.66** ± 0.08	**75.00** ± 3.27
Attention U-Net	98.59 ± 0.42	82.26 ± 2.55	86.02 ± 3.62	78.94 ± 3.55	99.46 ± 0.15	69.93 ± 3.69
RL + Attention U-Net	99.21 ± 0.16	84.56 ± 2.42	86.31 ± 2.90	83.54 ± 4.02	99.63 ± 0.08	73.31 ± 3.66
Residual U-Net	98.44 ± 0.61	80.56 ± 3.00	86.07 ± 4.20	75.96 ± 5.14	99.49 ± 0.12	67.53 ± 4.16
RL + Residual U-Net	99.19 ± 0.17	83.93 ± 2.34	86.07 ± 2.80	81.97 ± 3.42	99.65 ± 0.07	72.36 ± 3.51
TransUNet	98.42 ± 0.24	79.69 ± 5.02	81.73 ± 5.49	77.95 ± 6.49	99.26 ± 0.25	66.47 ± 7.00
RL + TransUNet	98.95 ± 0.22	80.31 ± 4.22	81.87 ± 3.22	78.42 ± 6.33	99.55 ± 0.12	66.54 ± 6.84
RetiFluidNet + Random	98.42 ± 0.59	82.37 ± 3.35	83.28 ± 4.80	80.38± 4.78	98.99 ± 0.27	70.03 ± 4.39

Note: All values in the table are percentages.

**Table 4:** Comparison of performance metrics from a five-fold cross-validation test set: with RL-augmentation vs. without RL-augmentation. The values presented are the *p*-values from the *t*-test comparing the two scenarios. Significant results (p<0.05) are highlighted in bold.

	Accuracy	Dice	Precision	Recall	Specificity	Jaccard Index
UNet	**0.040**	0.497	0.904	0.480	**0.023**	0.496
UNet++	**0.031**	**0.043**	0.199	0.061	**0.032**	**0.049**
Attention U-Net	**0.006**	0.837	0.660	0.909	**0.045**	0.587
Residual U-Net	**0.014**	**0.011**	0.592	**0.032**	0.051	**0.011**
TransUNet	**0.013**	0.182	0.357	**0.009**	**0.014**	0.184

## Data Availability

All relevant data, materials, and codes in this research are available upon request from the corresponding authors.
